# Promoting Maternal Health in the Postpartum Period to Advance Birth Equity

**DOI:** 10.3390/ijerph21121628

**Published:** 2024-12-06

**Authors:** Ariella Levisohn, Laurie Nsiah-Jefferson, Colette Dieujuste, Lisa Heelan-Fancher

**Affiliations:** 1 School of Medicine, Tufts University, Boston, MA 02111, USA; ariellabl@gmail.com; 2 McCormack Graduate School of Global and Policy Studies, College of Liberal Arts, University of Massachusetts Boston, Boston, MA 02125, USA; 3 Nursing Program, Wheaton College, Norton, MA 02766, USA; dieujuste_colette@wheatoncollege.edu; 4 Donna M. and Robert J. Manning College of Nursing and Health Sciences, University of Massachusetts Boston, Boston, MA 02125, USA; lisa.heelan-fancher@umb.edu

**Keywords:** birth equity, maternal health, postpartum, health equity, pregnancy

## Abstract

Black birthing people experience lower rates of postpartum follow-up care. The objective of this study was to examine factors associated with postpartum follow-up care and explore suggestions for improving the quality and experience of care during the postpartum period. A survey was conducted among Black birthing people in the Boston area who had delivered an infant within two years of the study. Our survey comprised the Jackson, Hogue, Phillips Contextualized Stress Measure (JHPCSM), the Power as Knowing Participation in Change Tool (PKPCT), and demographic questions. One hundred and twenty-one self-identified Black birthing people completed the survey. One-third of participants did not attend their postpartum appointment. Those with public insurance, an educational level of less than a college degree, or were working outside the home were significantly less likely to have a postpartum follow-up visit. Participants who attended postpartum visits had higher scores on the JHPCSM (lower stress) and PKPCT. Inability to take time off from work, COVID-19 concerns, and lack of childcare were the most frequently reported barriers to attending appointments. There is a need for better institutional and policy support for Black parents in the postpartum period.

## 1. Introduction

The postpartum period is increasingly recognized as the “fourth trimester” of pregnancy, starting after the birth of an infant and lasting for one year post-delivery. For many new mothers, this is an exciting time, yet for others, they face health concerns and challenges. In the United States, between 2017 and 2019, 53% of pregnancy-related deaths occurred between one week and twelve months postpartum [1]. The postpartum period is, therefore, an important opportunity to prevent maternal mortality.

Significant racial inequities exist in maternal health outcomes. Contributing to this is a complex web of factors that are ecological in nature and have an impact on utilization of postpartum care for Black birthing people. A review of the literature based on quantitative and qualitative studies highlights factors related to interpersonal, internalized, institutional [2,3,4], and structural racism [3], and socio-cultural factors that serve as barriers to the transition to postpartum care and hamper utilization of care for Black birthing people [5]. Within this context, in 2022, the overall rate of maternal mortality was 22.3 per 100,000 live births. Among non-Hispanic Black individuals, this number was 49.5 per 100,000 live births, over 2.5 times the rate among White birthing people. For non-Hispanic Black birthing people aged 40 and above, the rate was 174.5 per 100,000 live births [6].

Black individuals also have higher rates of underlying cardiovascular comorbidities prior to pregnancy compared to White individuals, and they are more likely to suffer from postpartum depression and hypertensive disorders in pregnancy, including gestational hypertension and preeclampsia [7]. Notably, the development of blood pressure-related conditions during pregnancy is an important predictor of cardiovascular complications later on in life, including atherosclerotic disease, hemorrhagic stroke, and heart failure [8]. Furthermore, while Black individuals may be less likely to develop gestational diabetes than other racial and ethnic groups, if they do receive a diagnosis of gestational diabetes, they have a higher risk of developing type 2 diabetes within the next ten years [9,10].

Postpartum follow-up visits are critical to identifying and addressing pregnancy-related complications before they result in maternal morbidity and mortality—for example, screening for high blood pressure to prevent progression to eclampsia—and they also offer a unique opportunity to prevent adverse health outcomes in the future. The American College of Obstetricians and Gynecologists (ACOG) recommends that everyone should have contact, either in person or virtual, with a maternal health provider within the first three weeks postpartum. For individuals with chronic or gestational hypertension, the ACOG recommendation is for follow-up within 3 to 10 days for a blood pressure check [11].

Despite these recommendations, postpartum visit attendance is below the Healthy People 2020 goal of 90.8% [12]. According to the Department of Health and Human Services (HHS) data, in 2016, 89.5% of people attended visits with a health care worker after giving birth. When examined by race, this percentage dropped to 86.0% for Black and African American postpartum individuals [12]. Furthermore, postpartum follow-up visits declined significantly during the COVID-19 pandemic, exacerbating these existing inequities. Although health care offices have seen postpartum visit rates begin to approach pre-pandemic levels, the rate of postpartum follow-up among Black individuals is rebounding more slowly than among White individuals [13].

Given the important role postpartum care can play in early identification and treatment of pregnancy-related complications and prevention of maternal morbidity and mortality, and the current inequities in maternal mortality, pregnancy-related complications, and access to postpartum care, it is necessary to better understand the perspectives and experiences of postpartum Black birthing people. The aims of this study were to (1) assess which demographic characteristics are associated with an increased likelihood of attendance at postpartum follow-up visits, (2) examine the associations between race and gender-specific stress (within the context of Critical Race Theory and feminism) and postpartum follow-up visits, (3) examine associations between power as knowing participation in change and postpartum follow-up visits, and (4) identify what Black birthing people perceived as barriers to accessing postpartum care during the COVID-19 pandemic.

## 2. Materials and Methods

### 2.1. Conceptual Framework

Critical race feminism and gendered racism are grounded in social justice and provide a framework to study racism and sexism, which are persistent and embedded in society [14]. Critical race feminism is concerned with how racism functions to produce social inequities at the intersections of race, class, and gender. The theory is an offshoot of Critical Race Theory (CRT) [15], as it emphasizes the multiplicative disadvantage of women of color as its distinguishing feature. First, Critical Race Theory espouses that racism is a ”normal and routine” part of American society [16]. Second, CRT employs storytelling to “analyze the myths, presuppositions, and perceived wisdoms common in American culture which denigrate black people” [16,17]. Third, CRT critiques liberalism, which addresses social challenges incrementally. Fourth, CRT highlights the colorblindness of laws and policies which ignore the different impacts they have on various racial/ethnic groups [17]. Fifth, CRT suggests that white elites will tolerate or encourage racial progress only if it promotes white self-interest [18]. Finally, CRT is skeptical of theories supporting meritocracy, ahistoricism, neutrality, objectivity, and single axis thinking [19]. These notions urge us to use various methods to highlight the counter stories by people of color to explain inequities in postpartum utilization, as well as to urgently interrogate and address the laws, policies, practices, and structures that affect inequities.

Furthermore, racism is a stressor for pregnant Black individuals [20,21]. This racial stress is linked with depression and adverse birth outcomes [22,23,24,25]. For Black women, additional exposure to stress over the life course may not only be due to racism, but both racism and sexism [26,27,28]. This type of stress—contextualized in Black women’s intersecting social identities—is known as gendered racial stress, and is generated by gendered racism, a hybrid form of oppression [27,29,30]. Gendered racial theory notes that Black women uniquely experience psychosocial stressors that go beyond reports of perceived instances of racism but also include sexism [31], and other forms of oppression, and may operate independently of other known sources of stress. The mechanisms in which gendered racism help to create these results include racially insensitive institutional care, the biomedical approach used in healthcare institutions, and unfair treatment based on health insurance and other factors, such as dismissed pain concerns because of the strong Black woman stereotype [5]. These realities reveal the experience of racial discrimination toward African American women through healthcare practices that are often seen as “standard” practices, albeit marginalizing minority populations. Furthermore, scholars have highlighted other threats to utilization, including mistrust of providers and institutions and worry about confirming and/or being judged by a stereotype. It has been found that gendered racism is more highly associated with reproductive stress versus general discrimination [20].

Barrett’s theory of power of knowing participation in change describes power in individuals and groups [32]. According to Barrett, every person is born with power; no one can give us power, and no one can take it away even if we are living in systems that are oppressive. Of course there are times that we may experience feelings of powerlessness, but Barrett states that there is no such thing as actual powerlessness [32]. In her theory, power is manifested through four inseparable dimensions: awareness; choices, even if the number of choices is limited and not ideal; freedom to act intentionally (from the available choices); and involvement in creating change. These four dimensions represent an individual’s or group’s power profile that is ever-changing, as power is not static and is about intentionally participating in change [32].

Yet Barrett’s theory contains two worldviews of power that exist side-by-side: power-as-control and power-as-freedom [32]. Power-as-control is a worldview that we commonly see every day—the laws of cause and effect. This type of power undergirds gendered racism and sustains it. Conversely, power-as-freedom represents a worldview in which there are no expectations or attachments to an outcome because people and their environment (which includes other people) are in continuous relationship; hence, none of us has full control. Nevertheless, everyone is engaged in change on some level whether we want to be or not, but the difference is whether we engage in change in a “knowing” or intentional manner. Therefore, Barrett’s theory of power was used to examine the relationship between a participant’s power profile and her involvement in creating change during the postpartum period.

### 2.2. Research Design

This cross-sectional correlation study comes from the quantitative component of a larger mixed-methods study. For this study, we examined the associations between and among race and gender-specific stress, power as knowing participation in change, and Black birthing people’s demographic characteristics and experiences of care during the postpartum period.

### 2.3. Sample

To be eligible to participate in our study, participants had to self-identify as Black, be between 18 and 45 years of age, have given birth to an infant (singleton birth) within two years of consenting to participate in our study, live in Boston or the Greater Boston area, and be able to read and speak English. Birthing people who did not meet these inclusion criteria were screened out and could not proceed with the survey. Our survey did not ask specifically about gender identity, so as such we use gender-neutral language such as “birthing people” and “parents” to refer to our sample. Prior to conducting this study, the sample size was estimated seeking a moderate effect size of 0.30, error (0.05), and power (1-*B* = 0.80). The power score suggested 82 participants were needed for a two-tailed test.

### 2.4. Instruments

The 135-item online survey consisted of two instruments and an author-created demographic questionnaire. Scholars of intersectionality theory call for the use of multidimensional measures that explore experiences of racism and sexism together, without disentangling them [33]. Although empirical research on gendered racism is becoming more prevalent, only a few intersectional instruments for gendered racism exist to our knowledge. One such instrument is the Jackson, Hogue, Phillips Contextualized Stress Measure (JHPCSM). Given that the JHPCSM is an instrument that specifically measures gendered and racial stress and was developed in collaboration with a diverse group of Black women and validated for use in pregnancy [33], we selected this instrument for our study.

The JHPCSM is a 68-item Likert scale instrument (1 = strongly agree to 5 = strongly disagree) with 6 subscales (race/racism, work, burden [gendered and financial stress], personal history, coping/support, stress) that was designed to assess chronic intersectional racial and gendered stress [34]. We reverse-coded items on the survey as needed such that a lower score of 1 is a “negative” response and suggests higher stress and a score of 5 is a “positive” response and suggests lower stress [34]. Some examples of items on this instrument include “Racism is a problem in my life”, “I have to work harder than white women to earn the same recognition”, “I have experienced a lot of loss (death of family or friends) in my life”, “I have limited access to credit”, “Because I am a woman, my employer is not usually open to suggestions from me”, “My religion or spirituality helps me to love myself”, and “I feel that I am alone”. The items for this instrument were derived from exploratory factor analysis, grounded theory, content analysis, and post-measure interviews with a sample of 474 African American women residing in Georgia. Cronbach alphas for the subscales ranged from 0.66 to 0.80 [34]. The Cronbach alpha for the total instrument is 0.84 [31].

The Power as Knowing Participation in Change Tool (PKPCT) is a validated instrument developed by Dr. Elizabeth Barrett to measure power profiles. The original PKPCT had 48 scored items, but due to Cronbach alphas being high (0.93 to 0.99), a shortened PKPCT with 12 items was created. In our study, we used the shortened version of the PKPCT. The PKPCT (short version) is a semantic differential scale that ranges from 1 to 7 and has 12 bipolar adjectives (items) under the question: “In relation to ____”. Some examples of the adjectives used were “unpleasant to pleasant” and “valuable to worthless”. Within the instrument there are four subscales: awareness, choices, freedom to act intentionally, and involvement in creating change; however, only the total instrument score was used for our data analysis. A higher score on the PKPCT suggests a higher power profile [32].

In addition, we created a 55-item demographic questionnaire. Examples of items on our questionnaire included sociodemographic characteristics of race, age, history of medical conditions, and socioeconomic items such as highest level of formal education, type of insurance, employment history, being a recipient of food stamps or WIC, and if they had seen a doctor or healthcare provider after the birth of their baby.

### 2.5. Recruitment

Recruitment took place using online formats such as Facebook and support groups, the University of Massachusetts Boston Birth Equity website, churches, and word of mouth. We also contacted over 200 women’s and women of color organizations in the Greater Boston area, WIC programs, and state and local government leaders. In June 2021, a town hall meeting was jointly held between the University of Massachusetts Boston and State Senator Liz Miranda, a champion for birth equity in Massachusetts, to further educate community members about birth inequities and inform them of this study.

### 2.6. Data Collection

Data were collected from April 2021 to January 2023. Notably, all of our participants gave birth and completed this study during the COVID-19 pandemic, which began in spring 2020 and officially ended in the United States on 11 May 2023 [35]. A 135-item survey was completed by participants via Qualtrics. No identifiable data were collected.

### 2.7. Data Analysis

We calculated descriptive statistics for participant characteristics and the JHPCSM instrument (total and 6 subscales) and PKPCT (total) scores. We conducted bivariate and multiple regression correlations to examine the associations between the JHPCSM instrument, PKPCT, and demographic characteristics. We used chi-square tests to examine the relationships between categorical variables, and independent sample *t*-tests to determine differences in sample means between those who attended postpartum follow-up visits and those who did not. An alpha level of 0.05 was used to examine statistical significance. We used IBM SPSS 27.0 for all statistical analyses.

### 2.8. Ethical Considerations

Ethical approval was obtained from the University of Massachusetts Boston Institutional Review Board. Our participants received written and verbal information about the study before consent was obtained. Participants were compensated for their time to answer the survey ($50 e-gift card). Additionally, they were informed that their participation was voluntary, and that the data would be treated confidentially.

## 3. Results

One-hundred and twenty-one self-identified Black birthing parents completed the online survey. Most of our participants were born in the United States (*n* = 90, 74%), had one or two children (*n* = 105, 87%), were married (*n* = 87, 72%), and had completed college or had earned a higher degree (*n* = 84, 69%). The most frequently represented age group was 25 to 29 (*n* = 49, 41%) (Table 1).

Overall, 61% of our participants indicated that they attended their postpartum follow-up appointments, while 34% of our participants did not keep or have a follow-up appointment after delivering their infant. Six declined to answer the question. Participants who reported a history of hypertension (*n* = 16) or diabetes (*n* = 8) prior to pregnancy reported the lowest rates of postpartum follow-up care (50%), and those who were diagnosed with gestational hypertension (*n* = 18) or gestational diabetes (*n* = 8) reported postpartum follow-up rates of 88% and 72%, respectively.

### 3.1. Factors Associated with Higher Rates of Attending Postpartum Follow-Up Appointments

Higher levels of formal education and private insurance were both significantly associated with higher rates of postpartum follow-up (Table 2). Among participants whose highest level of formal education was a 4-year college degree, 77% followed up postpartum. In comparison, only 46% of participants with less than a college degree followed up (*p* < 0.05). In addition, 77% of participants with private insurance attended their postpartum appointments, compared with only 55% of participants with public insurance (*p* < 0.05). Participants who did not work outside the home were more likely to attend their follow-up postpartum appointments than those who worked outside of the home (80% vs. 56%, *p* < 0.001).

There was no difference in rates of postpartum follow-up between modes of delivery (cesarean section vs. vaginal), birth weight (low birth weight, normal birth weight, high birth weight), or term (preterm, term, post-term). Additionally, postpartum follow-up rates were not associated with any differences in whether participants were currently using contraception. There were also no statistical differences in postpartum follow-up visits noted between US born and foreign-born participants. Although healthcare workers had higher rates of postpartum follow-up, the difference was not statistically significant.

### 3.2. Relationships Between Gendered Racism, Gendered Stress, and Postpartum Care

Participants’ total scores on the Jackson, Hogue, Phillips Contextualized Stress Measure (JHPCSM) ranged from 93 to 303 (*M* = 206.9). The scaled score (summation score) has a range of 68 to 340. Participants’ total JHPCSM scores were significantly associated with postpartum follow-up (*p* < 0.001); participants who attended their postpartum visits had overall higher scores on the JHPCSM instrument, indicating lower levels of racial and gendered stress. Additionally, when stratified by each JHPCSM subscale—race, burden, personal history, work, coping, and stress—the association with postpartum follow-up remained statistically significant (*p* < 0.05).

### 3.3. Relationships Between Power as Knowing Participation in Change and Postpartum Care

Participants’ scores on the Power as Knowing Participation in Change tool (PKPCT) ranged from 26 to 83 (*M* = 56.3). The scaled score (summation score) has a range of 12 to 84. Two participants did not complete the PKPCT portion of the survey. Participants’ PKPCT scores were also significantly associated with postpartum follow-up (*p* = 0.032). Participants who attended their postpartum appointments were more likely to have higher power scores.

### 3.4. Barriers to Postpartum Follow-Up Care

Fear of COVID-19 was the most common reason that participants did not follow-up with postpartum care (Table 3). Commonly cited concerns about COVID-19 included fear of infants getting the virus, especially if the infant or parent needed to have an appointment that was located within a crowded hospital or if they needed to take public transportation to get to an appointment, and inability to avoid exposures for those working in-person. Many participants also reported difficulties accessing medical services during COVID-19, with some participants finding it challenging to schedule appointments for themselves and their infant. Most individuals appreciated the availability of telehealth for the opportunity to avoid possible COVID-19 exposure, even though some also noted that it felt less effective than an in-person visit.

After fear of COVID-19, the second most selected reason for not attending postpartum appointments was an inability to take time off from work, with many reporting significant financial stress during the postpartum period. A few participants reported reductions in work hours, although it is unclear whether this was due to the COVID-19 pandemic, managers reducing work hours while employees were on parental leave, or whether the new mothers themselves requested fewer hours. Some participants also noted that their doctor’s office was far from their home or job, and so would require taking off multiple hours to attend an appointment.

The third most common response for why participants did not go to their postpartum follow-up visit was that “I wanted to, but I didn’t have anyone available to look after my children”. Many participants also noted childcare as an area of concern elsewhere on the survey, particularly finding and paying for childcare.

## 4. Discussion

In our study, we found that one-third of Black birthing people did not attend their postpartum follow-up appointments. Factors associated with lower postpartum follow-up rates included lower levels of formal educational achievement, public insurance, and working outside of the home. Individuals who did attend postpartum follow-up visits were significantly more likely to report higher JHPCSM (lower stress) and PKPCT scores. The three most frequently reported barriers to postpartum care included the COVID-19 pandemic, an inability to take time off from work, and lack of childcare.

Increasing the percentage of people attending postpartum medical appointments was a Healthy People 2020 goal. In 2016, overall postpartum visit attendance in the United States was 89.5%, only slightly below the goal of 90.8%. However, Black and African American postpartum people still experienced lower rates of postpartum care, at 86.0% [12]. HHS data also show that rates of postpartum care are lower for birthing people with public insurance, younger than age 18, income less than 100% of the Federal Poverty Level, those with less than a college degree, and those who are unmarried [12].

Other studies show large variability in postpartum follow-up rates. A systematic review of postpartum visit attendance in the United States found that attendance rates ranged from 25.9 to 96.5%, with a mean of 72.1% [36]. About a third of the studies included in the systematic review found that White birthing people were more likely to attend postpartum visits compared to birthing people of any other race or ethnicity, and multiple studies found that Medicaid beneficiaries had lower rates of postpartum follow-up [36].

The COVID-19 pandemic further exacerbated existing inequities. For example, in Boston, Massachusetts, one study estimated that rates of postpartum follow-up dropped from 75.2% pre-pandemic to 41.7% between January and March 2020, and rebounded to 60.9% between April 2020 and November 2021. Among Black birthing people, attendance rates were 63.2% pre-pandemic, 39.3% in early 2020, and 56.4% later in the pandemic. Black birthing people also had higher cancellation rates both before and during the pandemic compared to White birthing people [13].

Although our study was conducted during the COVID-19 pandemic, it also took place during the rise of the Black Lives Matter movement and widespread protests against police violence perpetuated on people of color. Experience with police brutality has been linked to medical mistrust, especially among racial minority groups [37]. We must consider that in 2020, there may have been heightened distrust in the medical system and in American institutions more broadly given the police violence occurring nationally and locally. As these events happened during our study period, they may have affected our results, as well as our participants’ interest in partaking in the study.

### 4.1. Postpartum Follow-Up for Birthing People with Medical Conditions

Our study highlights relatively low rates of postpartum follow-up for birthing people diagnosed with gestational hypertension and gestational diabetes. Previous studies have similarly drawn attention to low rates of postpartum follow-up among individuals diagnosed with medical complications during pregnancy. In 2016, one study found that only 54.6% of people with gestational diabetes had an in-office postpartum follow-up visit. In addition, this study found that Black individuals and those with public insurance were less likely to follow-up [38], a similar finding to our study. Given the known association between these diagnoses and risk of future diabetes and cardiovascular disease, it is important that individuals who have pregnancy complications receive follow-up care.

Even more concerning is the low rate of follow-up for individuals with preexisting conditions such as diabetes and hypertension, a topic on which there has been limited research prior to our study. Our data suggest a need for better counseling and education to increase awareness of the necessity of monitoring symptoms and getting appropriate clinical follow-up, both for underlying medical conditions and those diagnosed during pregnancy. ACOG recommends creating plans for postpartum care during prenatal visits, including identifying the specific care team with whom the patient will follow-up for management of chronic medical conditions, and discussing the need for postpartum glucose screening for people with gestational diabetes and postpartum blood pressure checks for those with gestational hypertension [11].

For individuals with hypertension and diabetes, either preexisting or gestational, more work also needs to be done around improving challenges with insurance coverage and inability to find a clinician with whom to follow-up. While previous studies have shown associations between barriers to care and postpartum outcomes using claims data and electronic medical record reviews [39,40], our study specifically asked a cohort of Black parents to reflect on the largest barriers from their perspective. These challenges do not exist within a vacuum, but instead highlight structural barriers that prevent Black communities from accessing care. Structural racism and institutional policies such as the GI bill, redlining, and mass incarceration are inextricably linked to social determinants of health such as insurance status and transportation [41].

For parents without insurance, there may be opportunities to enroll in Medicaid coverage either during prenatal appointments, or while in the hospital after delivery. Additionally, as per the ACOG recommendations above [11], the prenatal time is an ideal time to arrange for follow-up plans for management of chronic conditions, either with primary care providers or obstetricians. For birthing people who may not have prenatal care, utilizing social services within the hospital can help connect them with insurance coverage and medical providers prior to discharge.

### 4.2. Power, Agency, and Racial Stress as Predictors of Postpartum Visit Attendance

The finding that higher scores on the JHPCSM (lower stress) and PKPCT instruments are associated with higher postpartum visit attendance points to the ways in which perceived agency and power to knowingly participate in change can contribute to improved overall health. Participants who felt that they were better able to cope with their circumstances and had more freedom to act intentionally and make informed decisions were more likely to attend postpartum visits.

Additionally, we found that participants who scored lower on the race and stress subscales of the JHPCSM questionnaire (indicating higher levels of perceived racism, stress, racial stress, and gendered racism) were statistically less likely to follow-up postpartum. These findings highlight how exposure to racial stress on both an individual and a structural level, such as through educational and employment opportunities, can contribute to postpartum care utilization. Pregnant and postpartum people who are surrounded by individuals, communities, and policies that support them, rather than create barriers to equitable opportunities, are better able to prioritize their postpartum health. Furthermore, our findings direct us to consider racism through an intersectional lens, highlighting how experiences of racism are different and compounded for those who identify as women, and may be particularly amplified during the pregnancy and postpartum periods. Taken together, lower levels of gendered and racial stress and increased power may act synergistically to promote better postpartum outcomes.

### 4.3. Addressing Barriers to Postpartum Care

The results of this study suggest that fear of COVID-19, inability to take time off from work, and childcare responsibilities were barriers to getting postpartum medical care during the COVID-19 pandemic. Work as a barrier to care is supported by the significantly lower rate of postpartum visit attendance among individuals who work outside the home. In addition, participants who indicated higher rates of work stress on the JHPCSM work subscale were less likely to attend postpartum visits. As measured on the JHPCSM, the gendered racism occurring within the workplace is contributing to postpartum stress. Furthermore, our study suggests that work may be a barrier to care for a variety of additional reasons that are compounded by workplace discrimination. These include having limited time to take off from a job, restrictive family leave policies, low hourly wage jobs for which participants could not afford to miss time, and jobs that are located far from hospitals or medical offices.

Existing research also supports the significant role the COVID-19 pandemic played in exacerbating pregnancy and postpartum stress among Black birthing people, with one qualitative study in Mississippi highlighting lack of social support, fear of the virus, and disruption of health care services as particular challenges for Black birthing people in the postpartum period [42]. Our study reinforces these findings that COVID-19 created, and worsened, barriers to postpartum care [13].

Although our study participants were conflicted about whether telehealth was an effective way of providing medical care, increased availability of postpartum telehealth appointments might remove some barriers to postpartum care, such as concern over illness exposure and travel time. Home visits are another opportunity for improving access to postpartum care. Home visiting programs and virtual opportunities for connecting with health professionals are areas gaining increasing state and federal attention. Most recently, on 23 August 2024, Massachusetts signed a large maternal health bill, An Act Promoting Access to Midwifery Care and Out-of-Hospital Birth Options. This legislation, among other things, mandates insurance coverage of postpartum depression screenings and creates a postpartum home visiting service [43].

Additionally, in February 2024, HHS awarded a collective $1.8 million to ten projects as part of the HHS Racial Equity in Postpartum Care Challenge. Many of these projects are aimed at increasing access to care postpartum by utilizing technology, including mobile and web-based apps for virtual screening, counseling, and education, and virtual platforms for monitoring diabetes and hypertension symptoms postpartum, that are accessible by both patients and providers [44].

### 4.4. Future Policy Implications

In 2022, Dr. Karen Scott and Dr. Dána-Ain Davis offered the “Building and Bridging Black Futures Beyond Birth Model: A 12-Step Black Woman-Person First Approach”. The approach stresses holistic and culturally affirming postpartum care, and critiques prior work that has stigmatized Black birthing people and placed the onus on individuals rather than on systems for improving racial inequities in postpartum health outcomes. This model directs healthcare providers and professional organizations to initiate more widespread institutional change and respond to social, structural, and political barriers to care to advance postpartum equity [4].

Furthermore, better access to doula services is one area that has potential to improve postpartum outcomes among Black birthing people. Research suggests that doula support can improve postpartum mental health both through fostering social connection and offering medical services [45,46]. On the policy level, Massachusetts has committed to expanding access to doula services. The recently passed Massachusetts maternal health law mandates Medicaid coverage of doula services up to 12 months postpartum, and MassHealth (Massachusetts Medicaid) has been working on enrolling more doula providers in Medicaid [43]. However, current data on the impact of doulas on postpartum visit attendance is limited, so future research should examine whether Black birthing people with doulas are more likely to attend postpartum visits with a clinician after delivery.

An additional recent policy advancement in Massachusetts is the Massachusetts Paid Family and Medical Leave (PFML) Act, which became effective in 2019, shortly before data collection for this study began [47]. According to one analysis of 2016–2019 data, postpartum follow-up rates have a significant positive correlation with more flexible and comprehensive state paid family leave policies. Additionally, among Medicaid beneficiaries, postpartum visit attendance was slightly higher among those living in states with strong paid family and medical leave compared to those living in states with minimal or no laws on paid family leave [48].

Similarly, shorter leave duration increases the risk of not attending postpartum follow-up visits, with those with lower incomes at the highest risk of being unable to attend the visit [49]. These findings suggest leave duration is important for postpartum care attendance, and that low-income families should receive further support to improve postpartum care attendance regardless of leave duration. PFML is therefore an important step in reducing some of the financial and work-related barriers to care identified by many of the study participants, and national adoption of more comprehensive family leave policies could improve postpartum visit attendance and health among Black parents. Given the lower use of paid family and medical leave among birthing people of color, it is critical that we develop strategies to support them in taking leave [50].

Furthermore, Medicaid coverage was shown to be a negative indicator of postpartum follow-up in this study. This highlights the role of poverty as a social determinant that challenges postpartum care utilization, and further suggests a need for stronger quality metrics and payment incentives for postpartum follow-up. Better data are also needed on statewide postpartum follow-up attendance and general awareness of the importance of postpartum care. Other evidence-based opportunities for reaching Medicaid and lower income enrollees include improving engagement with community-based organizations to enhance social resource referrals for patients, educational and media campaigns focused on postpartum primary care, and establishing multidisciplinary postpartum care clinics [51].

Finally, efforts to make pregnancy and postpartum healthcare spaces safe for Black birthing people are needed to achieve equity. These include diversifying the maternal health workforce, such as through diversity and inclusion medical pipeline efforts [51], and enhanced medical education focused on respectful care and cultural humility [52]. Most importantly, understanding and addressing the unique needs of the Black birthing population within the context of both institutional and social determinants of health and everyday experiences with gendered racism is critical to moving towards racial equity in pregnancy and postpartum outcomes.

### 4.5. Limitations

The main limitation of this study is selection bias. Participants self-enrolled in the study, which required them to have access to a laptop or phone and internet access to participate. In addition, the study participants tended to be more educated than the general population. Another limitation is that the survey only asked specifically about whether participants were diagnosed with gestational hypertension, but not preeclampsia or eclampsia. Our results may therefore underestimate the true percentage of participants that had blood pressure-related complications during pregnancy.

## 5. Conclusions

Our study highlights the impact of perceived power to knowingly participate in change and gendered racism on postpartum follow-up, and how fewer experiences with gendered racial stress may be associated with an increased ability to prioritize personal health in the postpartum period. Our findings also demonstrate the emergence of COVID-19 as a significant concern for Black birthing parents, acting to discourage them from attending postpartum visits. Initiatives like telehealth services and home visiting programs provide opportunities to address barriers to postpartum care. On the policy level, the maternal health bill and PFML law in Massachusetts and the HHS Racial Equity in Postpartum Care Challenge offer exciting new frameworks for increasing access to postpartum care, as do recommendations for analyzing the role of policies of professional organizations who wield substantial influence surrounding practice. In addition, efforts to diversify the healthcare workforce are critically important, as is considering the unique needs of Black birthing people who are affected by structural racism and racial and gendered stress when implementing policy. Furthermore, advances in state data collection and quality metrics for postpartum care are needed to further track and evaluate opportunities for improvement. The recent policy innovations in Massachusetts can also serve as examples for other state and national lawmakers looking to address maternal health equity.

This research was primarily conducted during the COVID-19 pandemic, and therefore all rates reflect postpartum care received within this context. Future research might investigate whether and how COVID-19 continues to affect postpartum care for Black birthing people. Additionally, our research raises concern about the lack of postpartum follow-up among individuals with preexisting diabetes and hypertension and pregnancy complications such as gestational diabetes and gestational hypertension. There is a need for more, larger scale data on postpartum follow-up for these populations given the risks these conditions pose to future health and the importance of the postpartum visit for mitigating future risk. Finally, with new policy efforts in Massachusetts to improve family and medical leave and increase access to doula services, future research might examine the impact of these policies on postpartum visit attendance and outcomes for Black birthing people.

## Figures and Tables

**Table 1:** Demographic characteristics of participants (*n* = 121).

Variable	Category	Frequency	Percentage (%)
Age	18–24	8	6.6
	25–29	49	40.5
	30–34	44	36.4
	35–39	17	14
	40+	3	2.5
Race	Black or African American	118	97.5
	Multiple	3	2.5
Ethnicity	Hispanic	7	5.8
	Non-Hispanic	108	89.3
	Don’t know/missing	6	5.0
Education	High school or GED	4	3.3
	Some college	19	15.7
	College	46	38.0
	Graduate degree or higher	38	31.4
	Technical course/certificate	14	11.6
Location	Urban/inner city	90	74.4
	Suburban	25	20.7
	Rural	6	5.0
Speak >1 language at home	Yes	20	16.5
	No	101	83.5
US born	Yes	90	74.4
	No	30	24.8
Work outside the home	Yes	79	65.3
	No	42	34.7
Number of children	1	61	50.4
	2	44	36.4
	3	15	12.4
	4	1	0.8
Relationship status	Single	34	28.1
	Married	87	71.9

**Table 2:** Description of participants who attended their postpartum health care visits (*n =* 74).

Variable	Category	Frequency	Percentage (%)*
Education	High school or GED, some college, completed technical course/certificate	15	45.5 **
	Completed 4-year college	34	77.2 **
	Completed graduate degree or higher	25	65.8 **
Location	Urban/inner city	58	68.2
	Suburban	12	50.0
	Rural	4	66.7
Speak >1 language at home	Yes	14	82.4
	No	60	61.2
US born	Yes	57	66.3
	No	16	57.1
Healthcare worker	Yes	10	47.6
	No	64	68.1
Work outside the home	Yes	42	56.0 ***
	No	32	80.0 ***
Relationship status	Single	16	51.6
	Married	58	69.0
Delivery	Vaginal	56	62.9
	Cesarean	18	72.0
Gestational age	Term (37–41 completed weeks’ gestation)	66	66.7
	Preterm (less than 37 weeks’ gestation)	2	50.0
	Post-term (after 42 weeks’ gestation)	4	57.1
Birthweight	5.8–8.13 lbs	54	62.8
	<5.7 lbs	12	63.2
	>8.14 lbs	5	83.3
Insurance	Private	44	77.2 **
	Public	16	55.1 **
	Out-of-pocket	14	48.3 **
WIC benefits	Yes	28	62.2
	No	46	65.7
SNAP benefits	Yes	31	63.3
	No	43	65.2
Current contraception	Yes	34	68.0
	No	36	70.6

* Frequency is reported as the percentage of participants within the demographic category who attended a postpartum visit. ** Significant *p* < 0.05. *** Significant *p* < 0.001.

**Table 3:** Barriers reported by participants who did not attend postpartum follow-up appointments.

Reason	Participants (*n* = 41)		Participants with Gestational Diabetes and/or Gestational Hypertension (*n* = 6)	
	Frequency (*n*)	Percent (%) *	Frequency (*n*)	Percent (%) *
I wanted to, but I didn’t go because I was afraid of COVID-19	16	39.0	5	83.3
I wanted to, but I couldn’t get the time off from work to go	14	34.1	1	16.7
I wanted to, but I didn’t have anyone available to look after my children	11	26.8	1	16.7
I wanted to, but I didn’t have a doctor or midwife I could make an appointment with	9	22.0	2	33.3
I wanted to, but I didn’t have insurance	6	14.6	2	33.3
I wanted to, but I didn’t go because I didn’t have transportation to get there	5	12.2	1	16.7

* Note: participants could select more than one response.

## Data Availability

The datasets presented in this article are not readily available because participants gave consent on the grounds that survey and focus group data would be kept confidential and shared with only study team members.
